# The Personal Food Systems of Pre-Season NCAA Division 1 High-Contact, Low-Contact, and Non-Contact College Athletes

**DOI:** 10.3390/nu13113670

**Published:** 2021-10-20

**Authors:** Jennifer Peluso, Takudzwa A. Madzima, Shefali Christopher, Svetlana Nepocatych

**Affiliations:** 1 Department of Exercise Science, Elon University, Elon, NC 27244, USA; jpeluso@elon.edu (J.P.); tmadzima@elon.edu (T.A.M.); 2 Department of Physical Therapy Education, Elon University, Elon, NC 27244, USA; schristopher3@elon.edu

**Keywords:** contact, questionnaire, cross-sectional

## Abstract

Previous research indicates that dietary habits may differ between athletes of different sports. In this cross-sectional study, we hypothesize meal frequency, food choices, and food preferences will significantly differ between contact types. The participants were athletes (*n* = 92; men: *n* = 57, body fat percent (BF%): 14.8 ± 8.4%, body mass index (BMI): 25.5 ± 5.5 kg·m^−2^; women: *n* = 36, BF%: 26.7 ± 7.3%, BMI: 22.3 ± 2.7 kg·m^−2^) from high-contact (HCS), low-contact (LCS), and non-contact (NCS) sports. Meal frequency, food preference, and food choice questionnaires assessed factors influencing dietary habits. Dual-energy X-ray absorptiometry (DXA) measured lean body mass, fat mass, and body fat. A GLM multivariate analysis was used with significance accepted at *p* < 0.05. Significant body composition differences were observed between genders (*p* < 0.001) and among sports (*p* < 0.001). Dinner (83.7%), lunch (67.4%), and breakfast (55.4%) were the most frequently eaten meals, followed by evening snack (17.8%), afternoon snack (15.2%), and morning snack (8.7%). Greater preferences for starches were observed for HCS (*p* = 0.04; η^2^ = 0.07) and for a greater preference for vegetables was found for NCS (*p* = 0.02; η^2^ = 0.09). Significant differences also existed in the importance of health (*p* = 0.04; η^2^ = 0.07), weight control (*p* = 0.05; η^2^ = 0.11), natural content (*p* = 0.04; η^2^ = 0.07), and price (*p* = 0.04; η^2^ = 0.07). These results support our hypothesis that food choices and food preferences differ between contact types. This may help sports dieticians create more individualized nutrition programs.

## 1. Introduction

The American College of Sports Medicine, the American Dietetic Association, and the Dietitians of Canada agree that sport performance benefits should occur from following nutritional guidelines [1]. However, a majority of college athletes do not follow a diet optimal for sport performance [2] In fact, several studies have demonstrated that even high-level athletes often underestimate their daily needs [3,4]. Many athletes struggle to reach the recommended daily allowance (RDA) for adults, which have been suggested to be lower than athletes’ actual needs [3,5]. Thus, research is needed to provide coaches and dieticians with the right tools to provide clear and correct guidelines to athletes.

Personal food systems have been studied in different types of categories of athletes. For example, one study on interuniversity sports in India found differences in the macronutrient ratios of the diets of predominantly anaerobic sports and predominantly aerobic sports [6]. A study recording a 7-day food diary of the Australian Olympic teams found differences in endurance, team, sprint and skill-based, and weight-conscious sports [7]. Though research currently exists on the dietary food choices, preferences, and meal choices of certain sports, a gap exists in the literature in the comparison of food choices and preferences by contact level.

Contact types in sports are relevant to the world of sports nutrition studies because of the different belief systems that each person may hold as they pertain to their sport performance. This may affect body composition, which foods are preferred and avoided, and what times of day an athlete may eat. This introduction will describe each of the three studied contact levels: high contact sports (HCS), low contact sports (LCS), and noncontact sports (NCS). Additionally, hypotheses will be made regarding the body composition, preferences, and habits associated with each.

Contact level can be defined as the degree to which contact is integral to the sport. HCS allows high-impact, aggressive contact. HCS typically do not end without the athletes colliding, though there are exceptions. For example, we would still define ice hockey as a high contact sport because of the high number of physical fights that occur, though the fights are part of the culture of the game rather than necessity of game play. Stereotypes about HCS such as that they are primarily masculine sports and that athletes need to be large in order to overcome another individual during game play. We suspect that the aggressive and combative nature of HCS will affect the body composition, food choices, and preferences of these athletes. Football players represent the HCS group in this study. A previous study reported that over half of the football players in that study were actively trying to gain weight [8]. Smart and Bisogni (2001) state that building muscle mass while keeping fat mass low in order to “convey athletic power” was a focus of male college hockey players [9].

For the purposes of this study, we defined LCS as those in which athletes may come in contact with each other, but contact is not a goal of game play, and contact occurs as a consequence of other objectives such as stealing a ball in soccer or by blocking another player in basketball. Contact is allowed, though there may be rules that significantly limit the degree of contact, and the contact is not high-impact or aggressive in nature. Basketball, women’s lacrosse, and soccer represent the LCS group in this study. Women’s lacrosse is specified because women are not allowed bodily contact, but stick to stick contact is allowed, whereas in men’s lacrosse, there are no body contact rules. Since these sports have some bodily contact, being relatively large in mass is likely still important to these athletes.

NCS are completely contact free. Athletes never come into contact with one another because the sport is either individual (e.g., cross country) or opponents are separated by a physical barrier (e.g., a tennis net). Tennis and cross country running represent the NCS in this study. Having a large body mass for NCS athletes is not necessary, as there are no opponents to overcome. Additionally, in cross country running a low body mass index (BMI) is beneficial. Since moving against gravity is a major component, a lighter body mass equates to a more efficient use of energy while running [10]. It is hypothesized that this group will have the smallest body mass and will have the most concerns in terms of weight control. The goal of this article is to make a connection between contact level, body composition, food choices, and food preferences. We also analyze the meal frequency of the athletes participating in the study. We hypothesize that significant differences will exist between groups. The purpose of this study is to make sports dieticians and coaches aware of these differences so that they may be taken into consideration when preparing targeted nutrition programs.

## 2. Materials and Methods

### 2.1. Participants

This cross-sectional study was approved by the local Institutional Review Board: Elon University Internal Review Board (Protocol ID: #19-026). The study protocol was explained, and an informed consent form was signed prior to participation. A priori analysis designated a power of 0.8 and an alpha level of 0.05 and found the output total sample size to be 109 participants. A total of 125 NCAA Division I student-athletes were recruited from the local university for the study. Recruitment and data collection took place from July through August 2019 at the very beginning of pre-season training, as athletes were just arriving on campus. Thirty-three student-athletes were excluded from the study due to failure to complete either the questionnaires or the body composition assessment. Participant characteristics are provided in Table 1.

Athletes were categorized into HCS (*n* = 17), LCS (*n* = 51), and NCS (*n* = 24). HCS included football; LCS included men’s and women’s basketball, men’s soccer, and women’s lacrosse; NCS included men’s and women’s cross country running and women’s tennis. NCS are defined as sports in which athletes make no physical contact whatsoever; HCS are defined as sports in which the objectives of the game require heavy physical contact; and LCS are defined as sports in which physical contact is strictly regulated by rules that limit certain kinds of contact.

### 2.2. Anthropometric Data Collection

Height in meters and weight in kilograms were measured with a stadiometer (SECA, Chino, CA, USA) and balance beam scale (Detecto, Brooklyn, NY, USA), respectively, with athletes wearing light, athletic clothing and no shoes. Body Mass Index (BMI) was calculated as kilograms per meter squared. Body composition was assessed using dual energy X-ray absorptiometry (DXA) (General Electric, Boston, MA, USA) for lean body mass (LBM), fat mass (FM), and body fat (BF %). Participants were instructed to refrain from strenuous exercise, remain well hydrated, and fast for at least 8 h before the DXA procedure. Participants were positioned straight and centered on the table, and any objects (e.g., jewelry, watches, phones, etc.) affecting measurements were removed. Equipment calibration and quality control scanning procedures were performed daily.

### 2.3. Questionnaires

Qualtrics Survey Software (Qualtrics XM, Provo, UT, USA) was used to collect responses from the meal patterns (MPQ), food preferences (FPQ), and food choices questionnaires (FCQ). These questionnaires assessed eating patterns and factors influencing food choices and preferences.

### 2.4. Food Preference Questionnaire (FPQ)

The FPQ rated the liking of 62 foods on a six-point Likert scale ranging from “1, not at all” to “6, a lot”. Participants were instructed to select “not applicable” if they were not familiar with the food item. In addition, food allergies and dietary requirements were determined. Food preferences were categorized into six categories: vegetables, fruit, meat and fish, dairy, snacks, and starches. For example, participants would have rated beef burgers as part of the assessment for meat preference.

### 2.5. Food Choice Questionnaire (FCQ)

The FCQ provided insight into eating behaviors and food selection. Thirty-six food choices were categorized into nine categories including health, mood, convenience, sensory appeal, natural content, price, weight control, familiarity, and ethical concern. Each item was scored on a four-point Likert scale ranging from “1, not important at all” to “4, very important”. For example, participants may have rated the importance of food that is “easy to prepare” as part of the assessment for convenience.

### 2.6. Meal Pattern Questionnaire (MPQ)

The MPQ was used to describe meal frequency, type of meal, and time for meals in the past 28 days (4 weeks). Participants completed a seven-item questionnaire quantifying how often they ate breakfast, mid-morning snack, lunch, mid-afternoon snack, evening meal (dinner), evening snack, and nightly eating. Each item was scored on a seven-point Likert Scale from “0, no days” to “6, every day”. For example, participants may have selected having breakfast “1, 1–5 days” in the past 4 weeks [11].

### 2.7. Statistical Analysis

Descriptive data are presented as means and standard deviations. The Statistical Package for the Social Sciences version 22 software packages (SPSS, Chicago, IL, USA) was used to perform a general linear model (GLM) multivariate analysis with significance accepted at *p* < 0.05. Effect sizes (partial eta-squared: η^2^) were also used to determine the meaningfulness of the significant findings. Small, medium, and large effect sizes were described as 0.01, 0.06, and 0.14, respectively. Least Significant Difference (LSD) post hoc analysis was used to determine the differences between contact level sports when appropriate.

## 3. Results

Significant anthropometric differences were observed between genders (*p* < 0.001) and among all contact sport groups (*p* < 0.001). Refer to Table 1 for participant anthropometrics and characteristics between gender and sport. Gender and contact sport differences (*p* < 0.05) were observed for anthropometric characteristics; however, no significant differences were found between gender for the food preference (*p* = 0.81), choice (*p* > 0.05), with the exception of dairy (*p* = 0.04), or meal pattern questionnaires (*p* > 0.05). Women tended to consume more dairy than males. Therefore, males and females of the same sport and contact type were combined for FPQ, FCQ, and MPQ analysis.

Significant differences were observed in the preference for starches (*p* = 0.04) and vegetables (*p* = 0.02) with moderate effect size. HCS athletes had the highest preference for starches (4.3 ± 0.5) compared to NCS and LCS athletes, while NCS athletes had the highest preference for vegetables (4.1 ± 0.7) compared to HCS athletes. No significant difference (*p* > 0.05) was found in terms of preferences for fruit, snacks, meat and fish, or dairy. Overall, the preference for fruit was highest (4.3 ± 0.8) followed by snacks (4.3 ± 0.5), starches (4.1 ± 0.5), meat and fish (4.1 ± 0.7), and vegetables (3.8 ± 0.7). Dairy was the least preferred food group (3.8 ± 0.7). Please see Table 2 for FPQ results.

**Figure 1:**
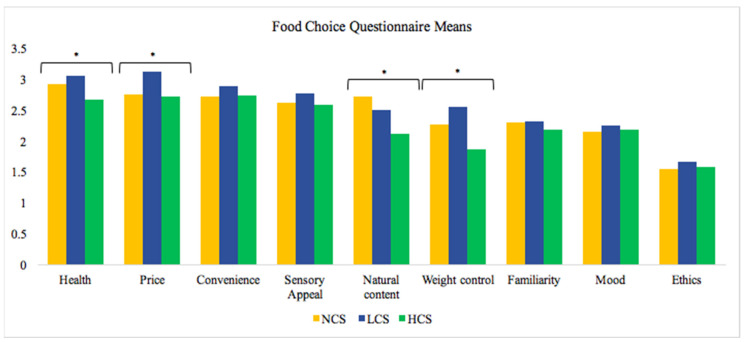
Food choice questionnaire results. NCS—noncontact sports, LCS—low contact sport, HCS—high contact sport. Note: * *p* < 0.05 represents a significant difference between groups.

Significant differences were observed in the importance of health (*p* = 0.05; η^2^ = 0.07), weight control (*p* = 0.01; η^2^ = 0.10), natural content (*p* = 0.04; η^2^ = 0.07), and price (*p* = 0.03; η^2^ = 0.07) with moderate effect size. LCS athletes placed the greatest importance on health (3.1 ± 0.4) and weight control (2.6 ± 0.7) compared to HCS athletes, whereas NCS athletes placed the greatest importance on natural content (2.7 ± 0.8) compared to HCS athletes. LCS athletes placed the highest importance on price compared to HCS and NCS athletes. No significant difference was observed in convenience, familiarity, mood, or ethics. Overall, the importance of health was the highest followed by price, convenience, weight control, familiarity, mood, and natural content. Ethics was the least important reason for choosing foods. Please see Figure 1 for FCQ results.

**Figure 2:**
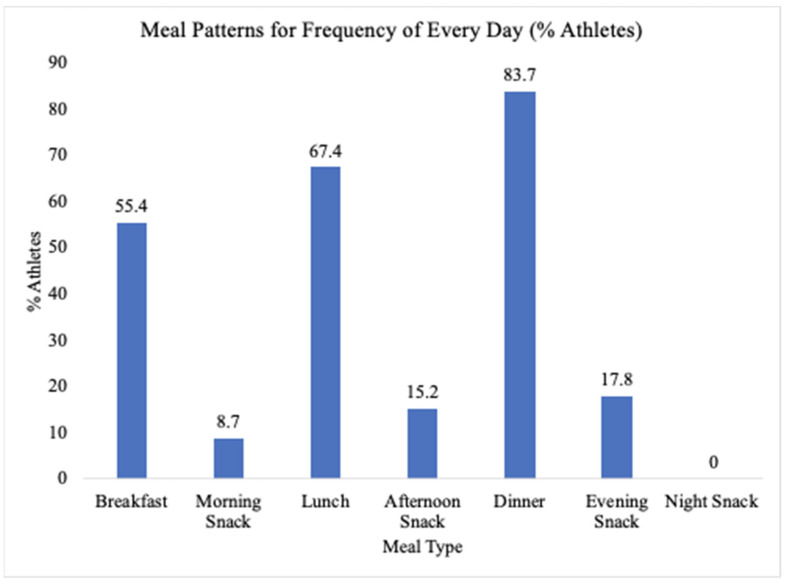
Meal pattern questionnaire results.

The meals that were the most frequently consumed every day were dinner (83.7%), lunch (67.4%), and breakfast (55.4%). Snacking in between meals was less common. Evening, afternoon, and morning snacks were consumed everyday by 17.8%, 15.2%, and 8.7% of the athletes, respectively. None of the athlete reported that they engaged in daily nighttime snacking. There were four NCS, four LCS, and two HCS athletes that answered “yes” to being a vegetarian or pescatarian. Please see Figure 2 for MPQ results.

## 4. Discussion

The purpose of this study was to assess pre-season body composition, eating patterns, food choices, and preferences in Division I NCAA college athletes. Considering that eating patterns and food choices impact body composition, these dietary habits may differ between contact types in sport. Finding no meaningful sex (Men v Women) differences in the MPQ, FPQ, and FCQ, the comparisons and discussion have been made based on differences in sport contact level.

Significant MPQ findings show that breakfast was the least consumed meal of the day for all of the athletes combined. Significant FPQ and FCQ findings show differences between groups were found for starches, vegetables, health, price, natural content, and weight control. Accounting for the likelihood of meaningful differences between athletes of different contact levels, dietitians may be better armed for individualized approaches to athlete nutrition.

### 4.1. DXA

The DXA results were expected. HCS athletes had the highest BMI, weight, and LBM followed by LCS athletes. NCS athletes had the lowest BMI, weight, and LBM. Interestingly, the three groups had approximately the same BF%, 19 to 20 percent. Another study shows differing results in that football players have a high BF% in comparison to other sports including basketball [12]. It is possible that gender differences skew the data here. All of the HCS athletes were male, while the NCS and LCS groups were mixed gender. The BF% of the HCS group is an average of 20.2 ± 10.9, while the average BF% of all of males in the study is 14.8 ± 8.4, and the average BF% of all of the females in the study is 26.7 ± 7.3. The study by Sanfilippo and colleagues (2019) supports the large standard deviation in the body mass of the football players, which is several points higher than that of either the NCS or LCS groups [12]. The study also found that football players had the largest variation in terms of body mass difference and linked the cause to the variety of physical requirements of each position [12].

### 4.2. Meal Frequency

The authors did not have adequate data to compare the meal frequencies of each contact type; however, the results from the MPQ are still of value and interest. These findings were included because studies show that meal frequency is an important component of a successful nutrition plan [13,14,15]. Our findings demonstrated that of the three main meals, breakfast was the least likely to be consumed daily, while dinner was the most likely to be consumed daily, followed by lunch. This is in agreement with other studies reporting that breakfast skipping was a common trend in NCAA Division I athletes [13,14]. Commonly cited reasons for skipping breakfast in the study by Shriver and colleagues (2013) were gastrointestinal discomfort with eating before workouts and poor availability of foods and beverages. Meals and snacks may be considered the most convenient in the evening, after classes and practices [14]. In addition, personal identities, including those who believe themselves to not be “breakfast people” may also play a small role [15].

However, consuming breakfast daily may be important for an athlete’s performance inside and outside of sport. The amount of glycogen stored in the liver and skeletal muscle is the primary factor determining energy availability, and low availability may lead to fatigue [16,17]. Taheri and colleagues (2019) found that male collegiate athletes showed significant increases in inhibitory control and performance on the Stroop Interference Test after consuming a breakfast of boxed cereal [18]. Low glucose availability may affect energy sources for the brain, decreasing cognitive function, and affecting performance in cognitive tasks [19].

Not eating breakfast has also been correlated with decreased training potential. Clayton and colleagues (2015) performed a study on male habitual breakfast eaters in which participants either did or did not eat breakfast and exercised 4.5 h after an ad. libitum lunch [20]. Though the breakfast skippers ate an average of 200 more calories during lunch, their total calories for the day were lower than those who ate breakfast. In addition, total work on the cycle ergometer was significantly lower for those who skipped breakfast [20]. However, the study is limited in that the participants were all habitual breakfast eaters; it cannot be concluded that those who are accustomed to skipping breakfast also experience the same decreased performance.

In reality, an athlete with an early morning workout is very unlikely to wake up several hours in advance for breakfast to impact sport performance. Studies have shown that ingesting carbohydrates in the form of drinks and bars during exercise promotes blood glucose and glycogen sparing, resulting in better exercise performance in comparison to no carbohydrate supplementation in running [21], cycling [22], tennis [23], and soccer [24].

On multi-practice days, meal timing for rapid muscle glycogen recovery and reduction of muscle-protein breakdown becomes especially important. Eating a meal immediately post-exercise may increase the rate of glycogenesis, preparing the body more quickly for another training session later in the day.

### 4.3. Food Preferences

Food preferences are considered to be affected by biological, social, and environmental factors [25]. While all humans have innate and learned inclinations and aversions towards certain foods, athletes have an increased social and environmental pressure to choose certain foods, which may subconsciously translate to food preferences [25,26].

The findings that HCS athletes preferred starches the most and that NCS athletes preferred vegetables the most was fairly consistent with the expectations of the authors. Abbey et al. (2017) states found that football players were consuming more starches, meat, and dairy products daily than fruits and vegetables [27]. Previous research supports our findings that HCS athletes prefer starches to vegetables. Considering a study that found that most football players are trying to gain weight [8], HCS athletes may have a higher preference for these foods due to their high energy density and carbohydrate content. However, athletes trying to lose weight have been shown to avoid carbohydrate rich foods, such as fruit and bread, because they believe that carbohydrates are responsible for weight gain [28].

It was surprising to find that HCS athletes did not have the greatest preference for meat/fish and dairy considering the emphasis on protein that other studies found. Abbey and colleagues (2017) noted in their study that protein powders were the most commonly used supplements by athletes and that a third of athletes consumed protein powders on a daily basis [27]. About 10% of athletes in this study followed a type of vegetarian diet, which may have influenced the results of meat and dairy preferences.

The preference for vegetables may be related to the finding that NCS athletes also placed more weight on foods that are of natural content. Cross country running is a weight-conscious sport [7]. Vegetables, due to their high fiber and water content, are typically low in calories and may help athletes maintain a lower weight without specifically eating for the purpose of weight-control [29]. However, the preference for vegetables may be more related to preference for foods with natural content rather than a way to maintain a relatively low body mass. Compared to other athletes, endurance athletes have shown higher caloric intake relative to mass [7].

### 4.4. Food Choices

Food choices are influenced by many factors, such as nutrition knowledge and beliefs, and social, psychological, and economic factors [26]. Athletes in particular are influenced by the advice from their coach, the practices of their peers, and the cultural norms of their specific sport [6,30]. Most athletes cite sport performance as the driving force of food choice, especially during competition season, in NCS, LCS, and HCS [31].

Health, natural content, and weight control were all significantly different between groups. In regard to the questionnaires of the current study, health-based choices were rated as the most important to athletes, and the athletes referred to choosing foods high in vitamins, minerals, fiber. The component “keeps me healthy” may be interpreted as providing the energy and strength to do daily activities. Similarly, natural foods are often perceived as a healthy choice. Natural foods were defined as having less additives and artificial ingredients. Health and weight control was most important to LCS athletes, while natural content was the most important to NCS athletes. It was surprising to the researchers that weight control was significantly more important to LCS athletes than it was for NCS athletes, considering that a theme in a study on female collegiate cross country runners included “I feel the less you weigh, the faster you run” [32] To an extent, research supports that body composition plays a role in performance outcomes in endurance sports such as cross country running [33]. However, being underweight is detrimental for sport performance—a well-known reason for this being the female athlete triad [32]. In choosing foods for weight-control, athletes prefer foods for their health and natural content qualities over their taste and convenience [34].

The price and convenience of food is related to an athlete’s access to food. Price was rated as the second most important consideration by all athletes in this study and was of the greatest concern to LCS athletes. Previous findings support the idea that the affordability of food is a top priority for college athletes [35]. The university attended by the participants provides on-campus dining as well as local restaurants and convenience stores. The majority of the incoming first-year students are required to have meal plans. Typically, student athletes self-determine their eating schedules and are limited by dining hall operation hours, sport practices, and their class schedule. The demanding training schedule takes time away that might otherwise be spent on going to a grocery store and cooking, which supports convenience as the 3rd most important consideration in choice [35].

## 5. Conclusions

In conclusion, our study supports the idea that differences exist in food preferences and food choices between HCS, LCS, and NCS athletes. Previous research findings suggest that sports belonging to these contact groups have different beliefs about what is the ideal body composition and diet for their sport. Clinically, the unique nutritional beliefs of athletes should be considered because it may pose challenges to meeting these needs, affecting health and performance outcomes. Sports coaches and dieticians may use this information to provide more targeted meal prescriptions and accommodate student athletes in need.

## 6. Limitations and Future Studies

Some limitations such as a small sample size and uneven distribution between contact sports should be considered when interpreting the results of the present study. The limited sample size required combining both men and women in the sport contact groupings, which is not ideal. In addition, we were unable to differentiate between positions in a single sport, which may have been substantial. The study had a power 0.8, and an α = 0.05 found the output total sample size to be 109 participants. We originally recruited 125 participants for this study, which would have been sufficient. However, we had to exclude 33 participants who did not submit complete surveys, resulting in a sample size of 92. Due to the limitations of the COVID-19 pandemic at this time, the collection of additional data is not feasible. Our main findings are not based upon the body composition of our participants but rather sport contact type. No gender differences were observed in the areas outside of body composition, except for preference for dairy. Thus, it was appropriate to group men and women together in the study based on sport contact type. Body composition is a point we have found interesting to discuss. We compared our findings to those of others. It is possible that gender differences may have affected the data. All of the HCS athletes in the present study were e male, while the NCS and LCS athletes were mixed gender. The BF% of the HCS athletes was an average of 20.2 ± 10.9, while the average BF% of all of the males in the study was 14.8 ± 8.4, and the average BF% of all of the females included in this study was 26.7 ± 7.3. Thus, it is possible that in reality, the HCS athletes did have a higher BF% than the other two groups, but this is not visible because the women in the study raised the averages of the group.

Retrospective dietary questionnaires such as the MPQ, FPQ, and FCP are recognized to be prone to subject bias. It is possible that the athletes reported what they thought the ideal meal patterns, food preferences, and food choices for an athlete should be. In future studies, a pre-familiarization trial is recommended.

Foods on the FPQ may not have been culturally appropriate for all student athletes. However, the MPQ was found to be acceptable for other studies [2], and the variety in each food category captured enough items to provide a mostly accurate idea of their preferences.

Further studies may choose to include questionnaires about performance goals and performance satisfaction, quantified by number of goals, wins, and times. Weight control might also include questions about choosing foods to put on weight, which may be applicable to HCS including football. Another relative comparison to make would be to compare the results of this study during pre-season to the results of this study during the competitive season. These additions may be helpful to coaches and dieticians to better understand the needs of athletes.

## Figures and Tables

**Table 1:** Participant Characteristics.

Measurement	Men (m)	Women (w)	NCS	LCS	HCS
*n*	57	36	24 (m = 8, w = 16)	51 (m = 32, w = 19)	17 (m = 17, w = 0)
Age (y) ^a,b^	19.5 ± 1.4	20.8 ± 1.3	21.1 ± 1.4	20.2 ± 1.3	18.2 ± 0.4
Weight (kg) ^a,b^	84.2 ± 17.0	65.0 ± 11.6	60.0 ± 6.8	78.2 ± 12.7	96.6 ± 19.3
Height (cm) ^a,b^	184.0 ± 8.3	171.0 ± 8.7	169.5 ± 6.5	181.8 ± 10.4	184.7 ± 6.3
BMI (kg·m^−2^) ^a,b^	25.5 ± 5.5	22.3 ± 2.7	21.2 ± 2.5	24.3 ± 5.0	28.2 ± 4.2
BF% ^a^	14.8 ± 8.4	26.7 ± 7.3	19.4 ± 9.3	19.3 ± 10.1	20.2 ± 10.9
LBM ^a,b^	67.1 ± 9.0	44.5 ± 5.0	46.0 ± 7.5	60.2 ± 12.3	71.2 ± 8.1

NCS—noncontact sports, LCS—low contact sport, HCS—high contact sport, y—years, kg—kilograms, cm—centimeters, kg·m^−2^—kilograms per meter squared, BMI—Body Mass Index, BF %—body fat percent, LBM—lean body mass; ^a^ *p* ≤ 0.05, significant difference between genders; ^b^ *p* ≤ 0.05 significantly different between HCS, LCS, and NCS.

**Table 2:** Food preference questionnaire results.

Category	Total Mean	NCS	LCS	HCS	*p* Value	η^2^
Fruit	4.3 ± 0.8	4.5 ± 0.6	4.2 ± 0.9	4.2 ± 0.7	0.25	0.03
Snacks	4.3 ± 0.5	4.3 ± 0.5	4.3 ± 0.5	4.2 ± 0.8	0.75	0.01
Starches *	4.1 ± 0.5	4.3 ± 0.6	4.0 ± 0.5	4.3 ± 0.5	0.04	0.07
Meat/fish	4.1 ± 0.7	4.0 ± 0.7	4.1 ± 0.7	4.0 ± 0.7	0.80	0.01
Vegetables #	3.8 ± 0.7	4.1 ± 0.7	3.8 ± 0.6	3.5 ± 1.1	0.02	0.09
Dairy	3.8 ± 0.7	3.9 ± 0.6	3.7 ±0.7	3.7 ± 0.8	0.46	0.02

NCS—noncontact sports, LCS—low contact sport, HCS—high contact sport. Note: * *p* < 0.05 represents a significant difference between LCS and other groups. # *p* < 0.05 represents a significant difference among all groups. η^2^ = partial eta-squared effect size.

## Data Availability

Not applicable.
